# Sperm Selection by Magnetic-Activated Cell Sorting before Microinjection of Autologous Oocytes Increases Cumulative Live Birth Rates with Limited Clinical Impact: A Retrospective Study in Unselected Males

**DOI:** 10.3390/biology10050430

**Published:** 2021-05-12

**Authors:** María Gil Juliá, Irene Hervás, Ana Navarro-Gómez Lechón, Fernando Quintana, David Amorós, Alberto Pacheco, Cristina González-Ravina, Rocío Rivera-Egea, Nicolás Garrido

**Affiliations:** 1 Andrology and Male Infertility Research Group, IVI Foundation—IIS La Fe Health Research Institute, Av. Fernando Abril Martorell, 106. Tower A, 1st Floor, 46026 Valencia, Spain; irene.hervas@ivirma.com (I.H.); ana.navarro@ivirma.com (A.N.-G.L.); nicolas.garrido@ivirma.com (N.G.); 2 IVIRMA Bilbao, Landabarri Bidea, 1–3, 2nd Floor, 48940 Leioa Bizkaia, Spain; fernando.quintana@ivirma.com; 3 IVIRMA Barcelona, Ronda del General Mitre, 14, 08017 Barcelona, Spain; david.amoros@ivirma.com; 4 IVIRMA Madrid, Av. del Talgo 68–70, 28023 Madrid, Spain; alberto.pacheco@ivirma.com; 5 IVIRMA Sevilla, Av. de la República Argentina, 58, 41011 Sevilla, Spain; cristina.gonzalez@ivirma.com; 6 IVIRMA Valencia, Pl. de la Policía Local, 3, 46015 Valencia, Spain; rocio.rivera@ivirma.com

**Keywords:** MACS, sperm, sperm selection, ICSI, cumulative live birth rate, pregnancy rate

## Abstract

**Simple Summary:**

Many couples attending infertility clinics still need to repeat treatments and undergo several failed attempts before achieving a healthy newborn, which leaves room for improvement in the techniques we currently use in the clinic. Among the different procedures susceptible to improvement, the selection of the most adequate sperm to be injected inside the egg is crucial to the cycle’s success. Magnetic-activated cell sorting (MACS) is a technique that removes physiologically abnormal sperm that have started a programmed cell death (apoptotic) process from a semen sample. However, it is not recommended to all patients because there is no agreement between the published literature on whether it improves reproductive outcomes. This study used data from all intracytoplasmic sperm injection cycles performed using the patient’s own oocytes in our clinics from January 2008 to February 2020. Our findings support that MACS should not be recommended to all infertile couples, since there was no significant difference in results compared to treatments in which MACS was not used. This study provides clinicians and patients with more accurate information on how MACS will impact their chances of pregnancy, and it will lead to studies focused on specific populations to which the technique can be particularly helpful.

**Abstract:**

The application of MACS non-apoptotic sperm selection in infertility clinics is controversial since the published literature does not agree on its effect on reproductive outcomes. Therefore, it is not part of the routine clinical practice. Classical measures of reproductive success (pregnancy or live birth rates per ovarian stimulation) introduce a bias in the evaluation of a technique’s effect, since only the best embryo is transferred. This retrospective, multicenter, observational study evaluated the impact of MACS on reproductive outcomes, measuring results in classical parameters and cumulative live birth rates (CLBR). Data from ICSI cycles using autologous oocyte in Spanish IVIRMA fertility clinics from January 2008 to February 2020 were divided into two groups according to their semen processing: standard practice (reference: 46,807 patients) versus an added MACS sperm selection (1779 patients). Only when measured as CLBR per embryo transferred and per MII oocyte used was the difference between groups statistically significant. There were no significant differences between MACS and reference groups on pregnancy and live birth rates. In conclusion, results suggest that non-apoptotic sperm selection by MACS on unselected males prior to ICSI with autologous oocytes has limited clinical impact, showing a subtle increase in CLBR per embryo transferred.

## 1. Introduction

Although the scientific literature provides embryologists and andrologists with morphological criteria to select the *a priori* most appropriate spermatozoon in the lab [1], this evaluation overlooks the unique molecular and genetic aptitude of each cell. Choosing an inadequate fertilizing sperm can lead to fertilization failure, incorrect embryo development, failed implantation, or miscarriage. Hence, sperm selection is crucial to ensure that the oocyte is correctly fertilized by the most competent [2]. Among the physiological properties involved in sperm function, apoptosis has been proposed as one of the more detrimental to spermatozoa’s fertilization potential. An increased presence of apoptotic markers activated caspase-3, externalized phosphatidylserine, or fragmented spermatic DNA has been linked to abnormal sperm motility or morphology [3,4,5,6,7,8], a decrease in fertilization rate and optimal quality embryos in couples with normozoospermic men [9], a decrease in fertilization potential, and a reduced ability to trigger acrosome reaction [10]. Therefore, the separation of sperm with initiated apoptosis is interesting to ensure the selection of the most physiologically competent sperm.

Magnetic-activated cell sorting (MACS) is a non-destructive cell separation technique that allows for the retention of apoptotic sperm cells expressing phosphatidylserine in their external membrane inside the column [5,9,11]. The eluted sample is enriched with non-apoptotic sperm, ready to be used in assisted reproduction technologies (ART) [12]. Despite not being performed routinely in the clinic, it is suggested to patients with high spermatic DNA fragmentation index, more than two unexplained intracytoplasmic sperm injection (ICSI) failures, and, in certain cases, more than two miscarriages with an unknown female cause. MACS combined with density gradient centrifugation (DGC) has been associated with a higher recovery of sperm with progressive motility (68%) when compared to neat ejaculate (39%), as well as lower DNA fragmentation index (4% MACS-DGC versus 24% in the reference) [3], and with improving the percentage of sperm with normal morphology [13]. In some studies, sperm selection via MACS showed a reduction of spermatic DNA fragmentation (fDNA) when compared to the neat ejaculate from asthenoteratozoospermic, teratozoospermic [14], and normozoospermic men [15]. However, one of these studies reported that the reduction of fDNA was not complete and not significant in all patients, since it was only substantial when samples had an initial fragmentation index ≥30% (7.1% after MACS versus 41.4% in the ejaculate) [16]. Another study reported no significant improvement in sperm morphology, motility, fDNA, or markers of fertilization capacity Izumo-1 and PLC-ζ comparing MACS combined with swim-up or DGC capacitation against controls [17]. Besides the effect it may have on enhancing sperm parameters, there is a significant lack of consensus on the extent to which sperm selection by MACS improves outcomes of standard ART cycles, as highlighted by recent meta-analyses [18,19].

Accordingly, this study aimed to retrospectively evaluate the effect of MACS sperm processing prior to ICSI in a large sample size to clarify the controversy surrounding its use. Reproductive success was measured by cumulative live birth rates (CLBR) per embryo transfer (ET), per embryo replaced, and per metaphase II (MII) oocyte until a live birth was achieved. By this approach, every embryo was considered a unique opportunity for pregnancy, providing a more realistic view of the impact of the intervention, eliminating the biases associated with measuring success in parameters that only contemplate the contribution of the best embryo in the cohort [20,21,22].

## 2. Materials and Methods

### 2.1. Study Design

This was a retrospective multicentric observational cohort study. Data were included from ICSI cycles using patients’ autologous semen samples and oocytes, performed at 15 Spanish IVIRMA clinics from January 2008 to February 2020, using semen samples from unselected males who underwent standard semen preparation (reference group) or an added sperm selection via MACS (study group).

### 2.2. IVF Procedures

Patient semen samples were collected, prepared, and examined as previously reported [23,24]. After this, capacitation via swim up [25] or density gradient centrifugation [26] was performed. Samples in the reference group were then used for ICSI, according to routine clinical practice. Samples in the MACS group were added with annexin-V-coated microbeads, incubated for their binding to apoptotic sperm with externalized phosphatidylserine, and processed through the column [25,26].

Ovarian stimulation and endometrial preparation were carried out as previously described [27]. Once ovarian follicles gained ≥17 mm in diameter, triggering was performed using recombinant human chorionic gonadotropin (hCG) or a single dose of GnRH agonist. Oocytes were retrieved 36 h after triggering and were then denudated [28]. After four hours, ICSI was performed and the resulting embryos were cultured [25], scored [29], and transferred. Pre-implantation genetic testing for aneuploidies (PGT-A) was performed on some of the embryos according to standard procedure [30]. Due to the vast time this study encompassed, ETs were performed either on day 2–3 of development or on day 5–6 at the blastocyst stage.

### 2.3. Database

An Excel database was created, containing information on patient and cycle characteristics as well as their outcomes. Prior to statistical analysis, an exploratory analysis was performed to identify outliers and discrepancies between the database and the exported data from the informatic platform used in the clinics. Data from 48,586 patients, 62,070 cycles, 389,212 embryos, and 500,260 oocytes were included.

### 2.4. Outcome Measures

The primary outcomes in this study were CLBR per ET, per embryo replaced (referring to the total number of embryos transferred to the same patient in consecutive cycles, not in the same transfer procedure), and per MII oocyte used in consecutive cycles until abandoning treatment or achieving a live birth. As commented in the introduction, this measurement was considered a more realistic approach to evaluate the effectiveness of a treatment or technique on the reproductive outcome. Reproductive success was also measured according to more classical outcomes: the biochemical pregnancy rate per ET, understood as the measure for beta hCG in blood serum higher than 10 IU/L at 14–16 days after ICSI, as well as the clinical pregnancy rate, the detection of a positive beta-hCG test result at 21–23 days after microinjection a week after a positive result in the biochemical pregnancy test, or confirmation via ultrasound of development of the fetal pole and heartbeat in weeks 6.5 to 7 of pregnancy. The ongoing pregnancy rate, the confirmation of the positive result of the clinical pregnancy test via ultrasound at week 12 of development, was also calculated per ET. The live birth rate (LBR) was calculated per transfer and per started controlled ovarian stimulation cycle, and the clinical miscarriage rate was expressed per transfer.

### 2.5. Statistical Analysis

All statistical analyses was performed using R version 4.0.0.

#### 2.5.1. Descriptive Analysis

A descriptive analysis was performed to study the behavior and distribution of variables referring to the patients’ and cycles’ characteristics and to evaluate the quality of the data and detect possible anomalies within them. For quantitative variables, the usual summary statistics where calculated, as well as 95% confidence intervals (95% CI) for the mean. Categorical variables were expressed as proportions. Means for the quantitative descriptive variables for both groups were compared by using paired *t*-tests to identify possible differences between the reference and the study groups, due to the retrospective nature of the study. For categorical variables, odds ratios (OR) were obtained and expressed with their 95% CI. The Chi-squared test was used to compare proportions. A *p*-value of <0.05 was considered statistically significant.

#### 2.5.2. Univariate Analysis

For the outcome rates per transfer and per cycle, Fisher’s exact test was used to compare both groups. For cumulative rates per transfer, per embryo replaced, and per MII oocyte consumed, Kaplan–Meier curves were plotted and compared via the Mantel–Cox test. A *p*-value of <0.05 was considered statistically significant.

#### 2.5.3. Multivariate Adjusted Analysis

A mixed-effects logistic regression model was developed to evaluate the association of variables of clinical impact in the main outcome, the ET resulting in a live birth or not. To correct the coefficient estimates of the fixed effects in the model, the patient identification number and the clinic in which the transfer was performed were chosen as random effects. The logistic model for live birth rate per cycle was adjusted for variables that were statistically significantly different between non-MACS and MACS groups, such as the age and BMI of the female patient, the presence or absence of male factor infertility determined by the semen samples’ conformance to the WHO 2010 guidelines for normality, and the transfer of the embryo at the blastocyst stage (over day 5 of embryo development), as well as variables considered of clinical relevance to the outcome based on previous experience of the group, such as the age of the male patient, last recorded endometrial lining, and whether or not the embryos of that cycle had been analyzed by PGT-A. Moreover, two separate models were created, dividing cycles into two populations: those who had the embryos analyzed by PGT-A and those who did not. To confirm the results, and control for potential confounders in the computation of CLBR, a Cox regression was performed considering the female patient’s age and BMI.

## 3. Results

### 3.1. Descriptive Variables

Summary statistics of the main characteristics of patients in both groups undergoing cycles that used the patients’ own oocytes are shown in Table 1. Since the same patient could have ART cycles performed with and without MACS sperm selection, descriptive variables were expressed per ovarian stimulation cycle. Female patients’ average age in the reference group (59,443 cycles) was 37.03 years (95% CI 37.00, 37.06) with a BMI of 23.22 (23.18, 23.25) kg/m^2^, while patients in the MACS group (2627 cycles) were 36.76 years (36.62, 36.91) and 23.05 (22.88, 23.21) kg/m^2^ on average. The average age for males was 38.88 (38.83, 38.92) years in the reference group and 38.77 (38.56, 38.97) years in the MACS group.

### 3.2. Main Outcomes: Cumulative Live Birth Rates

There were 49,350 cycles considered for the assessment of cumulative rates and plotting of survival curves, 47,235 in the reference group and 2115 in the MACS group.

**Figure 1:**
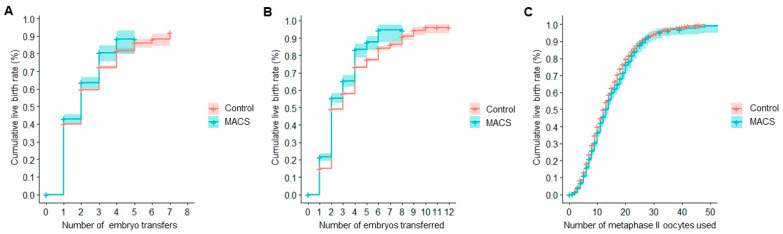
Cumulative live birth rates (CLBR) resulting from the unadjusted analysis of reproductive outcomes in cycles using autologous oocytes. (**A**) CLBR per embryo transfer. (**B**) CLBR per embryo replaced. (**C**) CLBR per patient’s own metaphase II oocytes used.

CLBR was first calculated per ET. For the MACS group, this rate was 43.0% (40.5%, 45.4%) for one transfer, 63.6% (60.1%, 66.8%) for two, 80.6% (75.5%, 84.6%) for three, and 88.3% (79.6%, 93.3%) for four, whereas the reference group presented a CLBR of 40.0% (39.5%, 40.5%), 59.6% (58.9%, 60.4%), 72.3% (71.2%, 73.4%), and 81.6% (80.0%, 83.4%), respectively. The plotted Kaplan–Meier curves are shown in Figure 1A. The Cox regression showed a statistically significant positive association between the processing of the semen sample through MACS (hazard ratio (HR) = 1.11, *p* = 0.009) and the CLBR per ET, which was consistent with the result obtained in the univariate analysis.

The CLBR per embryos transferred in the MACS group was 21.5% (19.4%, 23.6%) for one embryo, 55.5% (52.6%, 58.2%) for two, 65.4% (62.0%, 68.5%) for three, and 83.3% (78.9%, 86.7%) for four, while the reference group’s CLBR was 15.0% (14.7%, 15.4%), 49.1% (48.6%, 49.7%), 58.0% (57.3%, 58.7%), and 73.3% (72.5%, 74.1%), respectively. Kaplan–Meier curves are shown in Figure 1B. The difference between both curves was statistically significant. Consistent with the results of the univariate analysis, the Cox regression showed a statistically significant positive association between CLBR per embryo transferred and the use of MACS in semen processing (HR = 1.26, *p* < 0.001).

If computed per MII oocytes used, the reference group showed a CLBR of 13.1% (12.8%, 13.5%) for five MII, 39.8% (39.2%, 40.4%) for 10, 62.7% (62.0%, 63.4%) for 15, and 79.55% (78.74%, 80.32%) for 20 MII oocytes, while the MACS group had a CLBR of 11.0% (9.6%, 12.4%), 36.6% (33.9%, 39.1%), 59.8% (56.3%, 63.0%), and 76.25% (72.33%, 79.62%) for the same number of MII oocytes consumed. The Kaplan–Meier curves shown in Figure 1C were statistically significantly different. The Cox regression exhibited no significant relationship between the outcome and sperm selection through MACS.

### 3.3. Secondary Outcomes: Gestational Outcomes

When computed per ET, the MACS group had a 46.9% (45.2%, 48.7%) biochemical pregnancy rate, a 39.7% (39.4%, 40.0%) clinical pregnancy rate, and a 32.4% (30.7%, 34.1%) ongoing pregnancy rate, while the reference group showed 45.4% (45.0%, 45.8%), 38.5% (38.1%, 38.9%), and 31.8% (31.4%, 32.2%), respectively. None of these differences was statistically significant.

In terms of LBR, the MACS group showed a 29.3% (27.6%, 31.0%) per ET and a 38.8% (36.7%, 40.9%) per cycle. The reference group exhibited a 29.2% (28.8%, 29.6%) LBR per ET and 37.4% (37.0%, 37.8%) per cycle. Neither of the comparisons was statistically significant.

The MACS groups exhibited an 8.2% (7.1%, 9.3%) miscarriage rate per ET, whereas the reference group had a 7.5% (7.2%, 7.7%). The difference between groups was not statistically significant.

All gestational outcomes measured per transfer and, in the case of the LBR also per cycle, are displayed in Table 2.

When including selected clinical variables in the model, the adjusted OR for the association between the use of MACS and LBR per cycle was 1.02 (0.91, 1.14). The LBR per cycle in those where the embryos underwent PGT-A analysis showed an adjusted OR of 1.02 (0.84, 1.24), whereas in cycles in which embryos did not undergo PGT-A the adjusted OR was 1.02 (0.89, 1.17). None of these showed a statistically significant difference in LBR per cycle between the MACS group and the reference, as shown in Table 3.

## 4. Discussion

MACS sperm selection is currently not a part of routine clinical practice in fertility clinics. It is offered to patients in very particular cases with no standard treatment, such as men with a high fDNA index or couples with several failed cycles with no apparent female cause. Patients and clinicians tend to be willing to try diverse *add-ons* after several attempts have failed. However, their added costs should not be dismissed if their use is not justified by a proven increase in possibility to achieve a successful pregnancy [31,32]. Bearing in mind the controversy around the introduction of *add-ons* into the clinical practice without proper security and regulatory reviews [33,34], it is of utmost importance that clinicians are provided with reliable information resulting from carefully designed research, both prospective (randomized clinical trials or RCTs) and retrospective, making use of powerful statistical tools, proper designs, and bias control [35], to ensure that patients receive treatments catered to their needs and situation. This study aimed to determine whether the use of MACS results in an improvement in reproductive outcomes, measured as the number of oocytes, embryos, and transfer procedures required to obtain a live birth. Thus, it evaluated the true clinical impact of the enrichment of semen samples from unselected males with non-apoptotic sperm.

Concerning CLBR, cycles in the MACS group required a lower number of embryos to be transferred until a live birth rate was reached compared to the non-MACS group. Despite the MACS group needing a higher number of MII oocytes than the non-MACS group to obtain the same result, this difference was around 3.3% when consuming 20 oocytes, which, clinically, is meaningless for the patients in terms of increasing or lowering their possibilities to achieve a pregnancy. Results of the Cox regressions were consistent with the conclusions obtained from the univariate analysis: Even though the Mantel–Cox test showed a significant difference between Kaplan–Meier curves of the reference and MACS groups, the small difference observed (a 10.0% increase in CLBR when four embryos were transferred) was more likely due to covariates such as the female patients’ age and BMI rather than by the use of MACS during semen sample processing.

The use of MACS offered no improvement in pregnancy or live birth rates per transfer when compared to the reference group, as observed by the non-statistically significant differences between both groups of patients and cycles in their outcomes. Covariates such as the female patient’s age and BMI, the presence of male factor infertility, the last recorded endometrial lining, the transfer of the embryo after day 5 (at blastocyst stage), and the assessment of embryo ploidy via PGT-A could be influencing the correlation between the application of MACS as an added sperm selection step in semen sample preparation and the live birth rates per cycle. However, the adjusted OR of these associations are very close to 1, meaning that the size of the effect of the covariates on the live birth rate per cycle is, although significant, truly quite low.

The main limitation of this study was the broad reference population: unselected males in infertile couples. This introduced considerable heterogeneity between patients’ prognosis and etiologies, both between the two study groups (MACS and non-MACS) and within the reference group. As shown in Table 1, there was a statistically significant difference in terms of the proportion of patients labelled as ‘with male factor infertility’, which corresponds to a number of total count of progressive motile sperm lower than 5 million in the fresh ejaculate, and between the MACS (18.65%) and non-MACS (15.03%) groups. Even though the difference in absolute value was quite small, the authors acknowledge that the fact that MACS sperm selection is recommended in the clinic to patients with higher fDNA, several previously failed cycles and, overall, a worse prognosis introduce a bias in the comparison between these and cycles in which semen samples were processed following standard practice. The general scope that this study aimed to provide will progressively develop into separate analysis focused on different male and female etiologies and indications of the patients for undergoing ART, providing more information on the specific patient groups in which MACS could be more useful. As an example of a retrospective study focused on a distinct male population (>20% fDNA index) that could benefit from the use of MACS, Pacheco and colleagues recently reported a decrease in miscarriage rate and an increase in live birth rate when semen samples were processed via MACS versus the control group in cycles using autologous oocytes [36].

Incidentally, statistically significant differences between both groups in variables such as age and BMI were observed. Due to the vastly large amount of similar data that the study handled, any small difference between the groups would be picked up as significant by the analysis, also known as overfitting. Clinically, a difference in 0.2 years and 0.24 kg/m^2^ is not a meaningful disparity between these women. Nevertheless, these variables were controlled for during the statistical analysis.

Regarding the level of evidence provided by this reproductive study, it is worth noticing that RCT on this topic considered 29 [37], 138 [25], and 18 [38] patients in their MACS study groups and could not determine statistically significant relationships between the performance of MACS to the semen samples and their outcome variables (clinical pregnancy or miscarriage rates) [19]. In this study, which considered data from 389,212 embryos, 500,260 oocytes, and 62,070 cycles, differences between study groups as slight as a 6.4% in CLBR for two embryos transferred and 2.1% for five MII oocytes used were detected as statistically significant. This level of evidence, even if targeting a heterogeneous population, cannot be understated when drawing conclusions about infertile patients overall.

There is discrepant evidence on the effect of MACS sperm selection on clinical outcomes. One RCT showed increased pregnancy rates per cycle from 24.2% in the reference (DGC) group to 54.5% in the MACS-DGC, even though there was no significant improvement of fertilization or implantation rates, in couples with men factor infertility and at least two of the semen parameters below WHO 2010 normalcy criteria [18,36]. A similar RCT reported an increase of around 21% in LBR when using MACS compared to standard ICSI in normozoospermic men [38]. However, both RCTs had methodological issues, mainly incomplete outcome data or unclear randomization methods [19]. In another example focused on ICSI outcomes, even though the MACS group showed a 67.7% of good quality blastocysts while the standard ICSI group exhibited a 44.2%, there were no significant differences in LBR between groups [39].

Our results agree with the limited clinical impact of the use of MACS reported by previous studies: no improvement of ongoing pregnancy rates [13] and LBR in the MACS group in couples with idiopathic infertility using patients’ own semen samples [40], in unselected males in an ovum donation program [25], or in patients with a high level of spermatic DNA fragmentation [41], all of them undergoing ICSI. A meta-analysis by Gil and colleagues suggests that MACS, when compared to standard sperm selection, offers a slight improvement in pregnancy rates, though this does not translate into higher implantation or lower miscarriage rates [18]. The Cochrane database was not able to emit a clear conclusion on the effectiveness of MACS [19]. This lack of consensus could be due to differences in patient inclusion and exclusion criteria, the reduced number of patients recruited and then followed until the end of each cycle and pregnancy, or differences in semen sample processing techniques in each clinic. This may prevent meta-analyses to reliably compare results between studies [18,39].

## 5. Conclusions

Considering the largest sample size for these types of studies to date, our findings suggest that the separation of non-apoptotic sperm by MACS prior to ICSI in cycles in which autologous oocytes were used reduces the number of embryos required to be transferred in order to obtain a live birth when compared to the control group, although this difference seemed not clinically meaningful. As shown by the pregnancy rates and LBR measured per ET, the clinical impact of the selection of non-apoptotic sperm from samples from unselected males through MACS before performing an ICSI had no clinical effect on reproductive success measured in both classical parameters and CLBR. Even if the method itself is economically, practically, and logistically feasible, its application in fertility clinics cannot be justified unless its use provides a clear improvement in ART outcome rates.

## Figures and Tables

**Table 1:** Summary statistics for the main descriptive variables between the reference (semen samples processed according to routine clinical practice) and magnetic-activated cell sorting (MACS) groups in cycles using the patients’ autologous oocytes. The data show the mean and the 95% CI, as well as the *p*-value obtained using a *t*-test for the quantitative variables. For the categorical variables (*), results are expressed in proportions with their corresponding 95% CI and the *p*-value was computed using the chi-squared test.

Variable	Reference	MACS
Patient’s age (years)	37.03 (37.00, 37.06)	36.76 (36.62, 36.91)
Patient’s BMI (kg/m^2^)	23.22 (23.18, 23.25)	23.05 (22.88, 23.21)
Semen age (years)	38.88 (38.83, 38.92)	38.77 (38.56, 38.97)
Duration of sterility (years)	2.38 (2.36, 2.40)	2.53 (2.46, 2.60)
Number of oocytes retrieved	10.03 (9.97, 10.08)	11.43 (11.16, 11.69)
Number of MII oocytes	8.05 (8.01, 8.10)	9.03 (8.81, 9.26)
Number of available embryos per cycle	5.88 (5.85, 5.92)	6.50 (6.33, 6.68)
Number of viable embryos per cycle	2.37 (2.35, 2.39)	2.35 (2.25, 2.45)
Number of non-viable embryos per cycle	3.52 (3.49, 3.54)	4.16 (4.01, 4.30)
Days of ovarian stimulation (days)	10.66 (10.65, 10.68)	10.77 (10.70, 10.84)
Dose of gonadotropins (IU)	2247.82 (2240, 2256)	2159.57 (2124, 2195)
Estrogen level at day of ovulation induction (pg/mL)	1757.53 (1747, 1768)	1327.38 (1990, 2093)
Progesterone level at day of ovulation induction (ng/mL)	0.56 (0.44, 0.68)	0.76 (0.21, 1.31)
Days of endometrial preparation (days)	16.14 (16.11, 16.16)	16.83 (16.74, 16.93)
Last recorded endometrial lining (mm)	9.55 (9.54, 9.57)	9.53 (9.47, 9.6)
Last recorded estrogen level (pg/mL)	1438.09 (1428, 1448)	1526.18 (1479, 1573)
Last recorded progesterone level (ng/mL)	2.52 (2.09, 2.95)	1.54 (1.01, 2.07)
Male factor (%) *	15.03 (14.75, 15.32)	18.65 (17.16, 20.14)
Transfer over day 5 (%) *	36.74 (36.41, 37.07)	48.62 (47.05, 50.19)

**Table 2:** Results from the unadjusted analysis of gestational outcomes in cycles using autologous oocytes. The proportions for each group and the odds ratio (OR) are displayed with their corresponding 95% CI. The proportions are also shown with the sample number, either transfers or initiated cycles, for each of the outcome measurements. The *p*-value was computed using the Fisher’s exact test.

**Per Transfer**	**Reference**	**MACS**	**OR**	***p*-Value**
Biochemical pregnancy rate (%)	45.42 (45.04, 45.81)	46.94 (45.15, 48.74)	1.06 (0.99, 1.15)	0.1085
	*n* = 63,128	*n* = 2961		
Clinical pregnancy rate (%)	38.48 (38.10, 38.86)	39.68 (37.92, 41.44)	1.05 (0.97, 1.13)	0.1956
	*n* = 63,128	*n* = 2961		
Ongoing pregnancy rate (%)	31.80 (31.43, 32.16)	32.41 (30.72, 34.11)	1.03 (0.95, 1.11)	0.4904
	*n* = 62,807	*n* = 2931		
Live birth rate (%)	29.20 (28.84, 29.56)	29.30 (27.62, 30.99)	1.01 (0.92, 1.09)	0.9154
	*n* = 60,503	*n* = 2802		
Clinical miscarriage rate (%)	7.45 (7.22, 7.67)	8.22 (7.11, 9.33)	1.11 (0.95, 1.30)	0.1715
	*n* = 52,218	*n* = 2336		
**Per Cycle**	**Reference**	**MACS**	**OR**	***p*-Value**
Live birth rate ^1^ (%)	37.40 (36.96, 37.84)	38.82 (36.74, 40.89)	1.06 (0.97, 1.16)	0.1907
	*n* = 47,235	*n* = 2115		

^1^ Cycles with all oocytes consumed. Cycles in which the result was not a live birth rate but had still cryopreserved embryos to use in future transfer were not included.

**Table 3:** Results from the multivariate adjusted analysis, accounting for the relationship between the use of magnetic-activated cell sorting (MACS) and the live birth rate (LBR) per cycle, once adjusted for the female patient’s age and BMI, the age of the male patient, the presence or absence of male factor infertility, the last recorded endometrial lining measurement, the transfer of the embryo at the blastocyst stage, and the fact that the embryos underwent pre-implantation genetic testing for aneuploidies (PGT-A). This table shows the adjusted odds ratio (OR) with its 95% CI, standard error, and *p*-value for each studied population, namely: all patients included in the database, those whose embryos underwent PGT-A and those who did not.

Population	*n*	Adjusted OR	Standard Error	*p*-Value
All	59,443 reference cycles	1.018 (0.91, 1.14)	0.059	0.767
	2627 MACS cycles			
PGT-A	18,710 reference cycles	1.020 (084, 1.24)	0.102	0.846
	974 MACS cycles			
No PGT-A	40,733 reference cycles	1.017 (0.89, 1.17)	0.070	0.810
	1653 MACS cycles			

## Data Availability

No new data were created or analyzed in this study. Data sharing is not applicable to this article.

## Abbreviation

ART	assisted reproduction technologies
BMI	body-mass index
CLBR	cumulative live birth rate
DGC	density-gradient centrifugation
ET	embryo transfer
fDNA	DNA fragmentation
hCG	human chorionic gonadotropin
HR	hazard ratio
ICSI	intracytoplasmic sperm injection
LBR	live birth rate
MII	metaphase II
MACS	magnetic-activated cell sorting
OR	odds ratio
PGT-A	pre-implantation genetic testing for aneuploidies
RCT	randomized clinical trial
WHO	World Health Organization
95% CI	95% confidence interval
